# Lignin *p*-Hydroxybenzoylation Is Negatively Correlated With Syringyl Units in Poplar

**DOI:** 10.3389/fpls.2022.938083

**Published:** 2022-07-22

**Authors:** Yaseen Mottiar, Shawn D. Mansfield

**Affiliations:** Department of Wood Science, University of British Columbia, Vancouver, BC, Canada

**Keywords:** 4-hydroxybenzoic acid, lignification, monolignol conjugates, lignin, plant cell walls

## Abstract

The lignin found in the cell walls of poplar fibres is decorated with ester-linked *p*-hydroxybenzoate moieties that originate from the participation of acylated monolignols in lignin polymerisation. Although little is known about the biological implications of these cell-wall constituents, it has historically been postulated that acylated monolignols might promote lignification in syringyl lignin-rich species such as poplar. However, cell-wall-bound *p*-hydroxybenzoate groups were negatively correlated with syringyl units in a collection of 316 unrelated genotypes of black cottonwood (*Populus trichocarpa*). Based upon this observation, several alternative hypotheses on the occurrence of lignin acylation are presented.

## Introduction

Lignin is a phenolic biopolymer found in vascular plants that plays important roles in water conduction, biomechanics, and defence (Freudenberg and Neish, 1968). It occurs in the cell walls of xylem fibres, tracheids, and vessel elements along with cellulose, hemicellulose, and pectin. In wood and other lignocellulosic biomass, lignin can account for 20–35% of the total dry weight and is therefore extremely industrially important.

Lignin is assembled primarily from three cinnamic acid derivatives known as monolignols, namely *p*-coumaryl, coniferyl, and sinapyl alcohol, which give rise to, respectively, *p*-hydroxyphenyl (H), guaiacyl (G), and syringyl (S) units in polymeric lignin (Boerjan et al., 2003). The monolignols are produced in the cytosol *via* the shikimate, aromatic amino acid, and phenylpropanoid pathways, and are then exported to the cell wall where laccase and peroxidase enzymes catalyse the formation of resonance-stabilised phenoxy radicals that undergo radical coupling reactions to drive end-wise polymer growth (Ralph et al., 2004; Hoffmann et al., 2022).

Lignin is highly heterogeneous and non-canonical constituents occur in many species. Perhaps, most notable amongst these are the organic acids that are covalently linked *via* the γ position of monolignols (Ralph, 2010). For example, the lignins of commelinid monocots contain *p*-coumarate and ferulate groups (Harris and Hartley, 1980). Lignin is acetylated in kenaf, sisal, abaca, and hornbeam (del Río et al., 2007). And in poplar, willow, and several other species, *γ*-linked *p*-hydroxybenzoate moieties (henceforth denoted as *p*HB) constitute up to 5% of the lignin (Smith, 1955; Pearl et al., 1959; Goacher et al., 2021).

Since these organic acids are ester-linked to lignin, they can be released by simple mild alkaline hydrolysis. For this reason, they are known as ‘clip-off’ groups. There is considerable industrial interest in manipulating lignin acylation *via* genetic engineering or breeding since these clip-offs can potentially be separated from biomass and either used directly as platform chemicals or upgraded into more valuable bioproducts (Mottiar et al., 2016).

**Figure 1:**
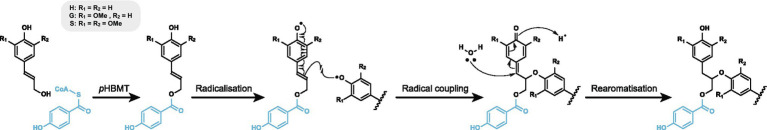
Reaction scheme showing the incorporation of *p*-hydroxybenzoate moieties (blue) into lignin *via* the formation of monolignol conjugates by *p*-hydroxybenzoyl-CoA:monolignol acyltransferase (*p*HBMT) and the participation of these conjugates in radical coupling.

It has been shown that ester-linked moieties arise *via* the incorporation of pre-acylated monolignols that participate in lignin polymerisation (Figure 1). Members of the BAHD superfamily of acyltransferases catalyse the formation of these monolignol conjugates. For example, monolignol acyltransferases responsible for conjugating monolignols with *p*-coumarate have been identified in rice, Brachypodium, and maize (Withers et al., 2012; Marita et al., 2014; Petrik et al., 2014). Homologous enzymes responsible for acylation with ferulate have also been reported in several monocot and dicot species (Wilkerson et al., 2014; Karlen et al., 2016; Smith et al., 2022).

Most recently, an enzyme involved in lignin *p*-hydroxybenzoylation was discovered in poplar, and downregulation of the corresponding gene significantly reduced lignin acylation (Zhao et al., 2021a; de Vries et al., 2022). We also recently developed a strategy to manipulate *p*HB levels for industrial gains by engineering the supply of *p*-hydroxybenzoate itself (Mottiar, 2021). Despite these advances, little is known about the biological implications of *p*HB groups or about the possible functions of lignin acylation more generally (Dixit and Nair, 2014; Yue and Lu, 2019). Herein, we show that *p*HB groups are negatively correlated with syringyl lignin in poplar even though they occur primarily in the syringyl-rich cell walls of xylem fibres and are bound to lignin *via* syringyl units.

## Materials and Methods

### Poplar Wood Samples

The samples analysed in this study were obtained from a collection of *Populus trichocarpa* genotypes grown in a common garden plot in Surrey, British Columbia. The 316 unrelated genotypes originate from across British Columbia, Washington, and Oregon, as described previously (Xie et al., 2009). Stem volume was determined using height and diameter measurements (McKown et al., 2014). Wood density was measured by X-ray densitometry of 10-mm increment core samples harvested at breast height from the north-facing side of 9-year-old trees (Porth et al., 2013). Any samples that exhibited signs of tension wood upon inspection using a hand lens were excluded from further analysis. Next, the core samples were disintegrated using a Wiley mill to pass a 40-mesh sieve. Wood extractives, including soluble phenolics, were then removed by exhaustively extracting the wood powder with hot acetone using a Soxhlet apparatus.

### Analysis of Lignin

The total lignin content was determined as the sum of the acid-soluble and acid-insoluble fractions resulting from a modified Klason lignin workflow, as described previously (Porth et al., 2013). Briefly, extractive-free wood powder was subjected to a one-hour swelling treatment in 72% sulphuric acid, followed by a 1-h acid hydrolysis reaction using 4% sulphuric acid at 121°C and 15 psi. The acid-insoluble fraction was determined gravimetrically as the residue retained upon filtering the hydrolysate through a medium-coarseness fritted glass Gooch-type crucible. The acid-soluble fraction was then measured spectrophotometrically at 205 nm using an extinction coefficient of 110 L g^−1^ cm^−1^.

The lignin composition (*i.e.,* % syringyl units) was evaluated by quantifying the monomers released from thioacidolysis, as described previously (Robinson and Mansfield, 2009). Briefly, extractive-free wood powder was subjected to thioacidolysis using 2.5% boron trifluoride diethyl etherate and 10% ethanethiol in freshly distilled dioxane for 4 h at 100°C. After neutralising with 0.4 M sodium bicarbonate, liquid–liquid extractions were performed with water and dichloromethane, and the reaction products were dried using anhydrous sodium sulphate. Finally, the released monomers were derivatised with *N*,*O*-bis(trimethylsilyl) acetamide and analysed by GC-FID.

### Analysis of Cell-Wall Polysaccharides

The amount of cellulose was measured using an alpha cellulose workflow, as described previously (Porth et al., 2013). Briefly, extractive-free wood powder was delignified by treatment with sodium chlorite and acetic acid. Non-cellulosic polysaccharides were then removed by extraction with sodium hydroxide. The remaining undissolved material was dried and weighed as cellulose.

The composition of cell-wall polysaccharides was evaluated by analysing the Klason lignin hydrolysate using HPAEC-PAD, as described previously (Porth et al., 2013). Xylose content was taken as a measure of the amount of xylan.

### Analysis of Cell-Wall-Bound *p*HB

The amount of cell-wall-bound *p*HB was measured by saponification following previously described methodology (Goacher et al., 2021). Briefly, extractive-free wood powder was subjected to alkaline hydrolysis in 2 M sodium hydroxide for 24 h at 30°C. The reactions were terminated *via* the addition of 72% sulphuric acid. Finally, the hydrolysates were filtered and analysed by reverse-phase HPLC-DAD. All samples were measured in duplicate, and the results were only included if the difference between technical replicates was less than 10%. In order to uncouple the extent of lignin acylation from the amount of lignin, cell-wall-bound *p*HB values (mg *p*HB/g extractive-free wood) were normalised by total lignin content (resulting in mg *p*HB/g lignin).

### Correlations Analysis

To ascertain the extent and directionality of pairwise correlations, the phenotypic datasets were analysed using SPSS Statistics 27 (IBM, Armonk, NY, United States). Although biomass, wood density, syringyl content, cellulose content, and xylose content followed a normal distribution, total lignin and cell-wall-bound *p*HB initially failed to pass the Shapiro–Wilk test for normality. Using the Box–Cox procedure, it was found that a simple logarithmic transformation of *x*′ = log(*x*) achieved normality for cell-wall-bound *p*HB, whereas a transformation of *x′* = (*x*^*λ*^−1)/*λ* with *λ* = 1.5 was appropriate for total lignin content. Finally, Pearson’s correlation coefficients were computed to assess pairwise correlations.

## Results and Discussion

It has been hypothesised that acylations such as *p*HB and *p*-coumarate could promote the formation of syringyl-rich lignin due to their propensity for radical transfer rather than radical coupling (Takahama et al., 1996; Hatfield et al., 2008). Indeed, compared to monolignols which feature a conjugated double bond on the propenyl sidechain, phenoxy radicals of *p*-hydroxybenzoate experience considerably less resonance stability. In addition, *p*-hydroxybenzoate lacks additional electron-withdrawing ring modifications (*i.e.,* hydroxys or methoxys) that would contribute to radical stability. In this way, the occurrence of *p*-hydroxybenzoate could theoretically allow for more radical transfers to sinapyl alcohol, and thereby accelerate the polymerisation of syringyl-rich lignin.

However, this hypothesis relies on the assertion that sinapyl alcohol is not a good substrate for peroxidases or laccases and, moreover, that *p*-hydroxybenzoate is a better substrate. There is evidence that *in vitro* dehydrogenative polymerisation of monolignols is slower when sinapyl alcohol is used (McDougall, 2001; Shigeto et al., 2018). However, class III peroxidases isolated from various plant species including poplar readily accept sinapyl alcohol (Sasaki et al., 2004; Marjamaa et al., 2009), as do some, but not all, laccases (Ranocha et al., 1999; Qin et al., 2020; Liu et al., 2021).

If indeed the function of *p*HB groups is related to sinapyl alcohol, one would reasonably anticipate that lignin acylation should be correlated with syringyl lignin units. Accordingly, wood samples representing a range-wide collection of *P. trichocarpa* were analysed. Cell-wall-bound *p*HB varied from 0.2 to 9.1 mg/g wood, representing 0.09 to 3.5% of the total lignin (see Supplementary Table S1). Syringyl content ranged from 62.8 to 79.2%, and total lignin content ranged from 15.5 to 25.7%.

**Figure 2:**
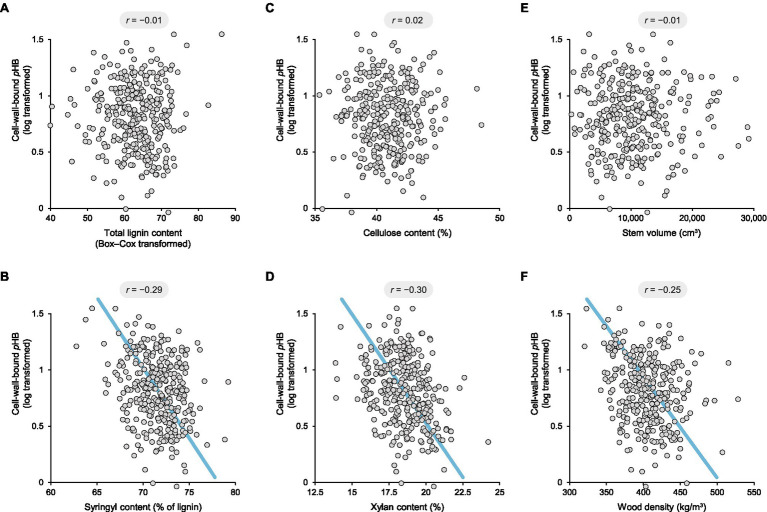
Scatterplots depicting pairwise correlations between cell-wall-bound *p*-hydroxybenzoate and total lignin content **(A)**, syringyl content **(B)**, cellulose content **(C)**, xylan content **(D)**, stem volume **(E)**, and wood density **(F)** in a collection of 316 9-year-old unrelated genotypes of *Populus trichocarpa* grown in a common garden. The *r* values provided above each plot are Pearson’s correlation coefficients. The blue lines depict reduced major axis regressions. Syringyl content, cellulose content, xylan content, stem volume, and wood density were normally distributed, whereas the datasets for cell-wall-bound *p*HB and total lignin content were transformed to meet the conditions of normality.

It follows that greater lignin content could potentially result in higher finite levels of *p*HB. Similarly, it is well known that lignin content and syringyl content are negatively correlated in poplar (Bose et al., 2009; Porth et al., 2013). However, the question at hand is whether *p*HB is specifically related to syringyl lignin units. Accordingly, cell-wall-bound *p*HB values were first normalised by total lignin content prior to analysis. It was then apparent that *p*HB levels were not substantially correlated with lignin content (Pearson’s correlation coefficient *r* = −0.01; Figure 2A).

On the other hand, a significant negative correlation—not positive—was observed between cell-wall-bound *p*HB and syringyl content amongst the 316 poplar genotypes (*r* = −0.29, *p* = 2 × 10^−7^; Figure 2B). Although unexpected, this observation is corroborated by a smaller study of 11 poplar genotypes, in which the *p*HB and syringyl contents were estimated by NMR analysis (Yoo et al., 2018, *r* = −0.63, *p* = 4 × 10^−7^). It should be noted, however, that NMR substantially overestimates lignin end groups since they have slower relaxation times than backbone structures (Mansfield et al., 2012).

The negative correlation with syringyl units is surprising for several reasons. Firstly, *p*HB groups are invariably attached to the γ position of syringyl rather than guaiacyl or *p*-hydroxyphenyl units in poplar. This has been shown using derivatisation followed by reductive cleavage (DFRC) reactions which sever β-aryl ether bonds but leave esters intact such that acylated monomers produce diagnostic markers (Lu et al., 2004), and by long-range two-dimensional NMR analysis using heteronuclear multiple bond correlation (HMBC) experiments (Stewart et al., 2009).

Enzyme kinetics analysis of the *p*-hydroxybenzoyl-CoA:monolignol acyltransferase from poplar showed that sinapyl alcohol is the preferred acyl acceptor substrate (Zhao et al., 2021a; de Vries et al., 2022). Indeed, all monolignol acyltransferases reported to date exhibit a preference for sinapyl alcohol *in vitro*. It has been shown that *p*HB groups are bound to syringyl units in palm species as well (Lu et al., 2015). However, there is at least one example in Nature where this is not the case. In the lignin of the Mediterranean seagrass *Posidonia oceanica*, *p*HB groups occur on both guaiacyl and syringyl units (Rencoret et al., 2020). Of course, since there are also many syringyl lignin-rich species that do not have acylated monolignols, they are certainly not a biochemical imperative.

One caveat with our analysis is that lignin acylation can impact the efficiency and yield of thioacidolysis. For instance, γ-acylation of syringyl units with *p*-coumarate reduced the cleavage of β-aryl ethers by as much as 40% (Grabber et al., 1996). We could reasonably anticipate that *p*-hydroxybenzoate would have a similar effect on thioacidolysis efficiency. Conversely, γ-acylation increases the proportion of β-aryl ethers (Lu and Ralph, 2008), the primary target of thioacidolysis, and could thereby counteract this effect by improving the overall yield. Accordingly, the occurrence of *p*HB groups can affect thioacidolysis in both positive and negative ways.

However, the proportion of syringyl units acylated with *p*HB ranged from just 0.07 to 2.35% (*i.e.,* far less than the overall variability in syringyl lignin content; see Supplementary Table S1). Similarly, we recently found that a 50% increase in *p*-hydroxybenzoylation of poplar lignin only altered the proportion of β-aryl ethers from 78.0 to 79.9% (Mottiar, 2021). Accordingly, it is highly unlikely that the observed correlations would be technical artefacts due to changes in thioacidolysis efficiency or yield caused by acylation.

The negative correlation with syringyl units is also unexpected given the distribution of *p*HB groups. We previously showed that *p*-hydroxybenzoylation occurs specifically in the cell walls of fibres and is largely absent from vessel elements (Goacher et al., 2021). Fibres in poplar contain a syringyl-rich lignin, and we observed that *p*HB groups spatially cluster with syringyl units. For these reasons, a positive correlation was anticipated. However, we also reported that cell-wall-bound *p*HB varied along developmental gradients in poplar stems and roots, and following nitrogen fertilisation.

To explore the possible connections between lignin acylation and cell-wall chemistry more broadly, we next looked at the cell-wall polysaccharides. Amongst the 316 poplar genotypes, cellulose content was as low as 35.3% and as high as 48.5%, whilst xylan content varied from 13.9 to 24.2%. There was no correlation between cell-wall-bound *p*HB and cellulose content (*r* = 0.02; Figure 2C); however, a significant negative correlation was apparent with xylan (*r* = −0.30, *p* = 4 × 10^−8^; Figure 2D).

Xylan plays diverse roles in plant cell walls, and it has been shown that the acylation of xylan with acetyl groups affects important interactions between cell-wall polymers (Busse-Wicher et al., 2014). The observation that *p*-hydroxybenzoylation correlates with xylan but not lignin nor cellulose contents could be an indication that lignin-bound *p*HB groups have an analogous role to xylan modifications. Indeed, it has been shown that lignin interacts primarily with xylan in poplar cell walls (Kirui et al., 2022), and it is known that lignin acylation impacts hydrophobicity and solubility by increasing the number of free phenolic groups (Lapierre et al., 2021). In addition, *p*-hydroxybenzoylation alters the structure of lignin by favouring β-aryl ether interunit linkages (Lu et al., 2015; Mottiar, 2021).

Finally, we also looked for possible correlations between lignin acylation and growth. Amongst the 316 poplar genotypes, stem volume ranged from 318 to 29,123 cm^3^ and wood density ranged from 320 to 528 kg/m^3^. Although there was no correlation between cell-wall-bound *p*HB and volume (*r* = −0.01; Figure 2E), there was a significant negative correlation with wood density (*r* = −0.25, *p* = 1 × 10^−5^; Figure 2F). This is most likely related to the proportion of vessel elements and the occurrence of *p*HB groups in the cell walls of xylem fibres rather than vessels.

Further investigations are needed before any specific biological roles can be ascribed to lignin acylation; however, some evidence has recently emerged that supports a role for *p*HB groups in cell-wall biomechanics. We reported that cell-wall-bound *p*HB is elevated in poplar tension wood (Goacher et al., 2021). It has also been shown that *p*-hydroxybenzoylation is involved in gravitropism (Zhao et al., 2021b). While it remains to be seen whether this is causative or merely correlative, trees with reduced *p*HB groups consistently exhibited growth and developmental defects.

Lignin acylations might also play a role in plant defence. In support of this hypothesis, cell-wall-bound *p*HB levels were increased in response to fungal and chemical elicitors in carrot (Schnitzler and Seitz, 1989), potato (Keller et al., 1996), tomato (Mandal and Mitra, 2007), and date palm (Latreche and Rahmania, 2011). However, *p*-hydroxybenzoylation was actually reduced in poplar wood upon wounding (Frankenstein et al., 2006).

**Figure 3:**
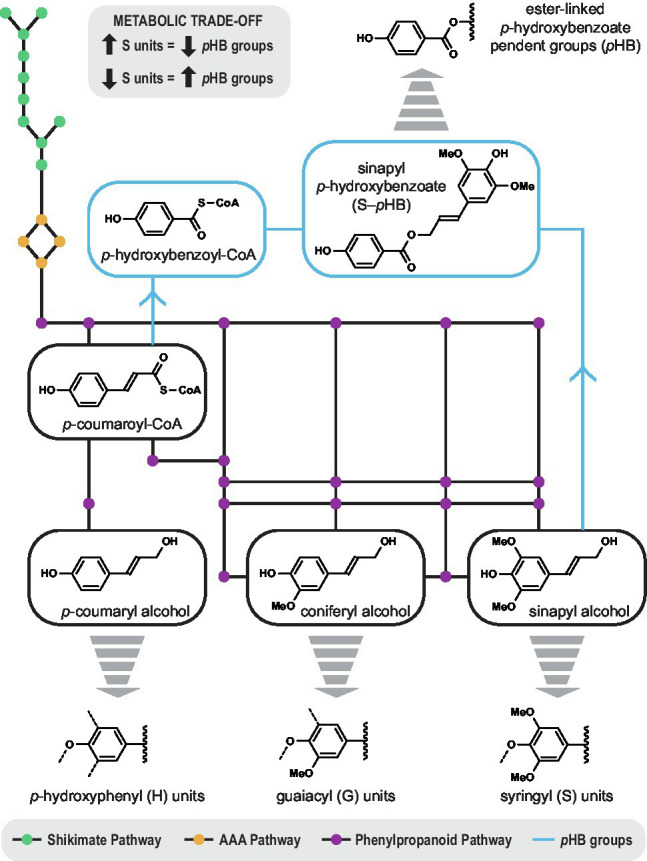
Biosynthetic map depicting the metabolic relationship between the formation of *p*-hydroxybenzoate groups (blue) and the monolignols *via* the shikimate pathway (green), the aromatic amino acid pathway (AAA, yellow), and the phenylpropanoid pathway (purple). *p*-Coumaroyl-CoA is a common precursor that links syringyl lignin units and *p*-hydroxybenzoate groups in a metabolic trade-off. A significant negative correlation was observed between cell-wall-bound *p*-hydroxybenzoate (from 0.9 to 35 mg/g lignin) and syringyl lignin (62.8 to 79.2%) amongst 316 unrelated genotypes of *Populus trichocarpa*.

Alternatively, it could be that *p*-hydroxybenzoylation of lignin is largely inconsequential for the plant, and merely a reflection of cellular metabolism (Figure 3). Since *p*-coumaroyl-CoA is a metabolic precursor to *p*HB groups as well as the monolignols (Terashima et al., 1975), perturbations in lignin biosynthesis intrinsically impact lignin acylation. For example, transgenic poplar with increased syringyl lignin units had lower levels of cell-wall-bound *p*HB (Stewart et al., 2009). Conversely, there was a decrease in syringyl units when *p*-hydroxybenzoate was increased by metabolic engineering (Mottiar, 2021). The negative correlation between *p*HB groups and syringyl units could be simply a manifestation of a metabolic trade-off. In this sense, perhaps, monolignol acyltransferases evolved as a way for plants to use up excess phenolics prior to programmed cell death.

It remains unclear what physicochemical changes accrue from *p*HB groups and, importantly, to what extent lignin acylation impacts plant development, biomechanics, defence, and physiology. However, we hypothesise that the metabolic relationship between the monolignols and *p*HB may be a driving force behind lignin acylation in poplar. Ultimately, a better understanding of lignin acylations could help inform future efforts to engineer plant cell walls, particularly those of syringyl lignin-rich biomass feedstock species.

## Data Availability Statement

The raw data pertaining to this study are provided in the Supplementary Material. Further inquires can be directed to the corresponding author.

## Author Contributions

YM collected the data and wrote the manuscript. YM and SM analysed the results, formulated the hypotheses, contributed to the article, and approved the submitted version.

## Funding

This work was supported by the Genome British Columbia Applied Genomics Innovation Program (award number 103BIO) and by the Great Lakes Bioenergy Research Center, United States Department of Energy, Office of Science, Office of Biological and Environmental Research (award number DE-SC0018409).

## Conflict of Interest

The authors declare that the research was conducted in the absence of any commercial or financial relationships that could be construed as a potential conflict of interest.

## Publisher’s Note

All claims expressed in this article are solely those of the authors and do not necessarily represent those of their affiliated organizations, or those of the publisher, the editors and the reviewers. Any product that may be evaluated in this article, or claim that may be made by its manufacturer, is not guaranteed or endorsed by the publisher.
